# Effects of traditional Chinese medicine exercise therapy on cancer-related fatigue, anxiety and sleep quality in cancer patients: A protocol for systematic review and network meta-analysis

**DOI:** 10.1097/MD.0000000000027681

**Published:** 2021-11-05

**Authors:** Lihao Jiang, Ju Ouyang, Xianfeng Du

**Affiliations:** Department of Oncology, The People's Hospital of Dazu District, Chongqing, China.

**Keywords:** anxiety, cancer, cancer-related fatigue, network meta-analysis, protocol, sleep, traditional Chinese medicine exercise

## Abstract

**Background:**

Cancer-related fatigue (CRF) is one of the most common adverse events of anticancer therapies, with an incidence of up to 90%, which seriously affects the quality of life in cancer patients. Complementary and alternative therapies for CRF include acupuncture, Chinese herbal medicine, Tai Chi, Qigong, and massage therapy. Several studies have shown that traditional Chinese medicine (TCM) exercise therapies, such as Tai Chi, Ba Duan Jin, the classics of tendon changing, Six Healing Sounds, and Wu Qin Xi, can improve CRF, anxiety symptoms, and sleep quality in cancer patients. However, their specific efficacy and which TCM exercise therapy is the best remain controversial. In this study, we assess and compare the effects of different TCM exercise therapies on CRF, anxiety, and sleep quality in cancer patients by network meta-analysis (NMA).

**Methods:**

Randomized controlled trials reporting TCM exercise therapies for CRF, anxiety and sleep quality in cancer patients published before October 2021 will be searched in the PubMed, Web of Science, Scopus, Cochrane Library, Embase, China Scientific Journal Database, China National Knowledge Infrastructure, Chinese Biomedical Literature Database, and Wanfang Data. Two researchers will be independently responsible for literature screening, data extraction and assessment of their quality. Standard pair-wise and NMA will be performed to compare the efficacy of different TCM exercise therapies on CRF, anxiety and sleep quality in cancer patients using Stata 14.0 software.

**Results:**

The results of this meta-analysis will be submitted to a peer-reviewed journal for publication.

**Conclusions:**

This NMA will contribute to identify the optimal TCM exercise therapy for CRF and provide evidence-based bias for clinical decision-making.

**Ethics and dissemination:**

Ethical approval was not required for this study. The systematic review will be published in a peer-reviewed journal, presented at conferences, and shared on social media platforms.

**OSF REGISTRATION NUMBER:**

DOI 10.17605/OSF.IO/MJ8DA.

## 1 Introduction

The American Association for Cancer Research in 2019 proposed that massive cancer research has driven a substantial progress with continuously decreased cancer-related death and increased survival rate.^[1]^ At present, quality of life and cancer-related symptoms in cancer patients have been well concerned.^[2]^ Cancer-related fatigue (CRF) is one of the most common and distressing symptoms in anticancer therapy, which is also the most prominent residual or persistent symptom in cancer survivors.^[3–5]^ The National Comprehensive Cancer Network defines CRF as a distressing, persistent, and subjective feeling of somatic, emotional, or cognitive fatigue or exhaustion that is inconsistent with recent activity levels, but related to the tumor or treatment of the tumor, and interferes with daily functioning.^[6]^ Clinical manifestations of CRF include weakness, cognitive impairment, drowsiness, mood disturbances, and low energy.^[7]^ Unlike the typical fatigue experienced by most people in the normal daily life, CRF cannot be relieved by rest or sleep.^[8]^

Up to 90% of cancer patients receiving antineoplastic therapy experience fatigue, and 27% to 82% of them remain fatigued after treatment.^[9]^ The high incidence and chronicity of CRF can seriously affect patient care and quality of life.^[10]^ The Guidelines for the Management of Cancer-Caused Fatigue published by the National Comprehensive Cancer Network recommend exercise therapy to manage fatigue symptoms in cancer patients.^[11]^

Traditional Chinese medicine (TCM) exercise therapy has been inherited and reformed with time, which is based on the holistic view of human life, and integrates TCM health preservation and care. The acceptable efficacy of TCM exercise therapy on strengthening the body and preventing diseases has been validated.^[12]^ In recent years, TCM exercise therapy has exerted a significant role in CRF treatment, which has been widely and flexibly used in clinical practice, and served as an important tool for effective prevention of CRF.^[13–17]^

Valid evidences have confirmed the efficacy of multiple TCM exercise therapies on CRF in cancer patients.^[18–21]^ However, it is unclear which one is optimal. In addition, different TCM exercise therapies have their own advantages and disadvantages. To our knowledge, a network meta-analysis (NMA) on comparing the effects of different TCM exercise therapies on CRF, anxiety, and sleep quality in cancer patients has not been reported. To promote the rational use of TCM exercise therapy and provide evidence-based basis, this study will conduct a NMA for analyzing randomized controlled trials (RCTs) reporting TCM exercise therapies on interfering CRF, anxiety and sleep quality in cancer patients.

## 2 Methods

### 2.1 Study registration

The protocol of this review was registered in OSF (OSF registration number: DOI 10.17605/OSF.IO/MJ8DA). This protocol was designed according to the guideline of Preferred Reporting Items for Systematic Review and Meta-analysis Protocols.^[22]^ The findings of this study will be reported in line with the guideline of Preferred Reporting Items for Systematic Reviews and Network Meta-analysis.^[23]^

### 2.2 Inclusion criteria for study selection

#### 2.2.1 Types of studies

RCTs reporting the efficacy of TCM exercise therapies on CRF, anxiety, and sleep quality in cancer patients.

#### 2.2.2 Types of participants

The diagnosis of cancer was confirmed by pathological or cytological examination, and CRF was diagnosed based on the diagnostic criteria.^[24]^ There will be no restriction on the gender, region, or race of participants.

#### 2.2.3 Types of interventions

In the experimental group, patients will be treated with TCM exercise therapies, such as Tai Chi, Ba Duan Jin, the classics of tendon changing, Six Healing Sounds, and Wu Qin Xi; while those in the control group will receive standard management without exercise interventions.

#### 2.2.4 Types of outcome indexes

i) CRF: Pipers’ Fatigue Scale, Multidimensional Fatigue Symptom Inventory, and Cancer Fatigue Scale scores;
ii) Anxiety: Self-rating Anxiety Scale and Hamilton Anxiety Scale scores;
iii) Sleep: Pittsburgh sleep quality index.

### 2.3 Exclusion criteria

1) Non-RCTs;
2) Editorials, letters, reviews, etc;
3) Duplicate publications;
4) Absence of complete data or full-text.

### 2.4 Data sources

RCTs reporting TCM exercise therapies for CRF, anxiety, and sleep quality in cancer patients published before October 2021 will be systematically searched in the PubMed, Web of Science, Scopus, Cochrane Library, Embase, China Scientific Journal Database, China National Knowledge Infrastructure, Chinese Biomedical Literature Database, and Wanfang. Searching strategy in the Pubmed was depicted in Table 1. The appropriate combination of MeSH terms, free words, and keywords will be searched in online databases.

**Table 1:** Search strategy in PubMed database.

Number	Search terms
#1	Neoplasms[MeSH]
#2	Cancer[Title/Abstract]
#3	Tumors[Title/Abstract]
#4	Benign neoplasms[Title/Abstract]
#5	Neoplasia[Title/Abstract]
#6	Neoplasm[Title/Abstract]
#7	Neoplasms, benign[Title/Abstract]
#8	Benign neoplasm[Title/Abstract]
#9	Cancers[Title/Abstract]
#10	Neoplasm, benign[Title/Abstract]
#11	Tumor[Title/Abstract]
#12	OR/1 to 11
#13	Exercise therapy[MeSH]
#14	Therapy, exercise[Title/Abstract]
#15	Exercise therapies[Title/Abstract]
#16	Therapies, exercise[Title/Abstract]
#17	Tai Ji[MeSH]
#18	T’ai Chi[Title/Abstract]
#19	Tai Chi[Title/Abstract]
#20	Tai Ji Quan[Title/Abstract]
#21	Tai-ji[Title/Abstract]
#22	Taiji[Title/Abstract]
#23	Taijiquan[Title/Abstract]
#24	Tai Chi Chuan[Title/Abstract]
#25	Chi, Tai[Title/Abstract]
#26	Ji Quan, Tai[Title/Abstract]
#27	Quan, Tai Ji[Title/Abstract]
#28	Traditional Chinese medicine exercise therapy[Title/Abstract]
#29	Ba Duan Jin[Title/Abstract]
#30	Classics of tendon changing[Title/Abstract]
#31	Six Healing Sounds[Title/Abstract]
#32	Wu Qin Xi[Title/Abstract]
#33	Qi Gong[Title/Abstract]
#34	Liu Zi Jue[Title/Abstract]
#35	OR/13-34
#36	Randomized controlled trials as topic[MeSH]
#37	Clinical trials, randomized[Title/Abstract]
#38	Controlled clinical trials, randomized[Title/Abstract]
#39	Trials, randomized clinical[Title/Abstract]
#40	Random^∗^[Title/Abstract]
#41	OR/36 to 40
#42	#12 AND #35 AND #41

### 2.5 Data collection and analysis

#### 2.5.1 Data extraction and management

EndNote X9 software will be used to exclude duplicate title information, merge the same literatures from different databases, create a database involving eligible RCTs and downloads their full text. Data extraction will be carried out independently by 2 researchers and cross-checked for review using a predeveloped form.

The following data will be extracted: basic information, including the first author, journal and year of publication, and title; RCT information in experimental and control group, including case number of each group and total number, age, intervention, duration of treatment, and outcome indicators; quality evaluation of the included literature; and outcome indicators.

**Figure 1:**
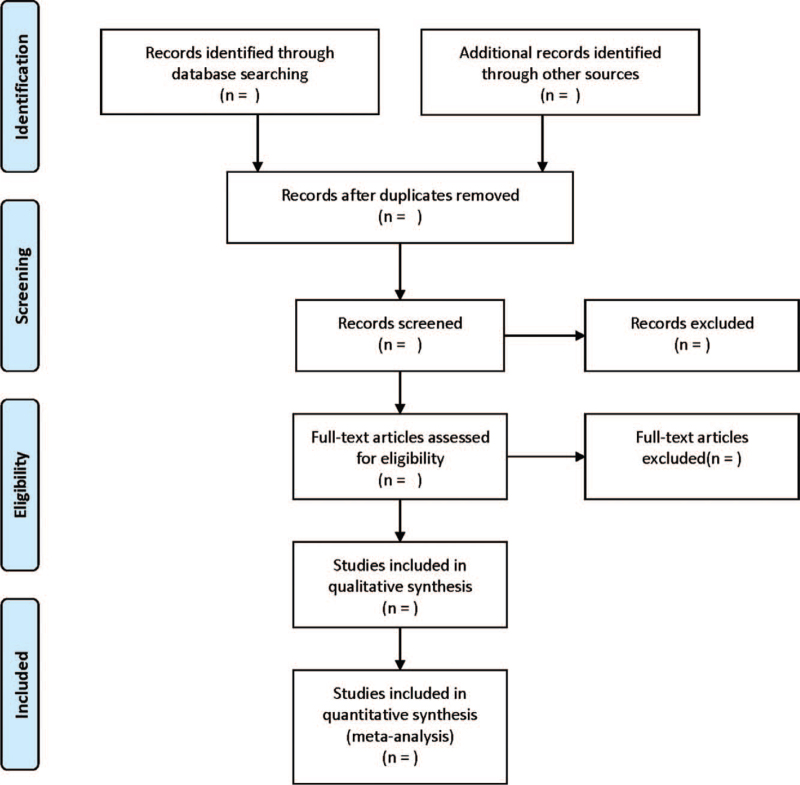
Flow diagram of study selection process.

The flow chart for literature screening was presented in Figure 1.

#### 2.5.2 Assessment of risk of bias

The methodological quality of included RCTs will be evaluated according to the Cochrane Risk of Bias Assessment Tool.^[25]^ The evaluation indicators will include the randomization method, allocation concealment, blinding, completeness of outcome data, selective reporting, number of dislodged cases, follow-up, and other biases. Each item will be subdivided into high risk, low risk, and uncertain risk. Based on the description of the above aspects by the included RCTs, 3 investigators will be responsible for determining and evaluating the methodological quality.

#### 2.5.3 Measures of therapeutic effect

The effect size of continuous variable data will be calculated with the standardized mean difference and corresponding 95% confidence intervals (CIs).

#### 2.5.4 Management of missing data

In case of any missing data in relevant study, the original data will be requested by E-mail; otherwise, they will be excluded from this study.

#### 2.5.5 Assessment of heterogeneity and data synthesis

STATA 14.0 software (STATA Corporation, College Station, TX) will be used to perform the pairwise meta-analysis and NMA. Chi-square test will be performed to measure the heterogeneity among the direct comparison results, and I^2^ will be conducted to measure the heterogeneity. If there is no heterogeneity (I^2^ < 50%, *P* > .1), a fixed-effects model will be adopted in the meta-analysis; otherwise, a random-effects model will be adopted.^[26]^ The STATA 14 software will be used to draw a network diagram to visually present the comparisons between all therapies.

The inconsistency factor and corresponding 95% CI will be calculated to evaluate the consistency of each closed loop. The value of 95% CI of the inconsistency factor value at 0 will indicate the consistency between direct and indirect comparative evidence, as well as the closure loop; otherwise, the closure loop will be considered inconsistency. After comparing multiple exercise interventions, the pooled surface under the cumulative ranking curve will be calculated for each intervention, and they will be ranked according to surface under the cumulative ranking curve.

#### 2.5.6 Assessment of reporting biases

Comparison-adjusted funnel plots will be plotted to analyze the existence of publication bias if 10 or more pieces of literature are included in this meta-analysis.^[27]^

#### 2.5.7 Subgroup analysis

Subgroup analysis will be performed based on the timing of the intervention.

#### 2.5.8 Sensitivity analysis

Sensitivity analysis will be performed by a one-by-one elimination method to verify the robustness of the results.

#### 2.5.9 Grading the quality of evidence

The evidence quality will be independently evaluated by 2 reviewers using the Grading of Recommendations Assessment, Development and Evaluation, which classifies the quality of evidence as high, medium, low, and very low.^[28]^

#### 2.5.10 Ethics and dissemination

The contents of this paper do not involve moral approval or ethical review and will be presented in print or at relevant conferences.

## 3 Discussion

CRF is a persistent, subjective exertional feeling in physiological, emotional or cognitive aspects related to tumor or tumor treatment, which is characterized by rapid development, heavy degree, high energy consumption and long duration, etc.^[29,30]^ It is a common clinical symptom in cancer patients, with an incidence rate as high as 96.5% and a degree of moderate to severe, which seriously affects patients’ quality of life.^[31]^ TCM exercise therapies are functional in strengthening the body and relaxing the mind, which is of significance in promoting cancer recovery. However, their specific efficacy on CRF, anxiety and sleep quality in cancer patients has not been compared. NMA is able to quantify and analyze different interventions for treating the same disease, while ranking all intervention strategies to determine the best intervention. This study will summarize and rank the effects of different TCM exercise therapies on CRF, anxiety, and sleep quality in cancer patients by comparing them through NMA, thus providing a reference for determining the optimal one.

## Author contributions

**Conceptualization:** Xianfeng Du, Lihao Jiang.

**Data curation:** Lihao Jiang.

**Funding acquisition:** Xianfeng Du.

**Investigation:** Ju Ouyang.

**Methodology:** Ju Ouyang.

**Project administration:** Xianfeng Du.

**Supervision:** Xianfeng Du.

**Validation:** Ju Ouyang.

**Visualization:** Ju Ouyang.

**Writing – original draft:** Xianfeng Du, Lihao Jiang.

**Writing – review & editing:** Xianfeng Du, Lihao Jiang.
